# Editorial: Host and Pathogen Determinants of Allergic and Invasive Fungal Diseases

**DOI:** 10.3389/fimmu.2020.00856

**Published:** 2020-04-30

**Authors:** Joshua J. Obar, Agostinho Carvalho, Joana Vitte, Stéphane Ranque

**Affiliations:** ^1^Department of Microbiology & Immunology, Geisel School of Medicine at Dartmouth, Lebanon, NH, United States; ^2^Life and Health Sciences Research Institute (ICVS), School of Medicine, University of Minho, Braga, Portugal; ^3^ICVS/3B's - PT Government Associate Laboratory, Guimarães, Portugal; ^4^Aix Marseille Université, Institut de Recherche pour le Développement, Assistance Publique-Hôpitaux de Marseille, Service de Santé des Armées, MEPHI: Microbes, Evolution, Phylogénie et Infection, Marseille, France; ^5^IHU Méditerranée Infection, Marseille, France; ^6^Aix Marseille Université, Institut de Recherche pour le Développement, Assistance Publique-Hôpitaux de Marseille, Service de Santé des Armées, VITROME: Vecteurs – Infections Tropicales et Méditerranéennes, Marseille, France

**Keywords:** fungal diseases, fungal infection, fungal allergy, allergic bronchopulmonary mycoses, aspergillosis, coccidioidomycosis, talaromycosis, cryptococcosis

Fungal diseases are mostly known as opportunistic acute life-threatening invasive fungal diseases, such as *Candida* blood stream infection, invasive aspergillosis, cryptococcal meningitis, *Pneumocystis* pneumoniae, histoplasmosis, and mucormycoses. But they also include chronic fungal infections that are defying treatment, such as mycetoma, sporotrichosis, fungal keratitis and chronic pulmonary aspergillosis, coccidioidomycosis, or histoplasmosis; and fungal allergic diseases, such as allergic bronchopulmonary mycoses, allergic fungal sinusitis, hypersensitivity pneumonitis, or atopic dermatitis. This high heterogeneity in the onset and clinical course of fungal diseases raises fundamental questions about their pathogenesis, which results from either a lack of recognition by the immune system or an excessive inflammatory response. The 10 articles of this themed collection highlight the latest advances regarding host and pathogen determinants of allergic and invasive fungal diseases.

Two critical reviews of the literature update the current state of knowledge on invasive fungal diseases and allergic mycoses. Firstly, Van Dyke et al. address the battle against coccidioidomycosis, a neglected disease whose incidence has raisen to 350,000 cases/year in the desert areas of the western United States. Their review details the current knowledge on the protective host immune responses, potential vaccines, and new treatments against *Coccidioides immitis*. Secondly, Hadebe and Brombacher address the excessive inflammatory response associated with fungal diseases, particularly in the setting of chronic disease. They summarize the current view on (i) early exposure to environmental fungal species; (ii) their contribution to the development of allergic responses; (iii) the mechanisms of host tolerance controlling immune hyper-responsiveness to ubiquitous fungi, and (iv) the scarce knowledge on the early factors involved in the development of fungal allergic diseases. These issues relate to the major unanswered question is why a given fungus (*Aspergillus, Alternaria, Cladosporium, Malassezia*, or *Penicillium*) will cause allergy or infection rather than be perceived as innocuous at a given time point in a given human host. Allergy to fungal molecules triggers allergic bronchopulmonary mycoses, allergic sinusitis, allergic bronchitis, hypersensitivity pneumonitis, and atopic dermatitis. Fungal molecules with established allergenic potential have been previously described, but the tools for *in vitro* or *in vivo* diagnosis of fungal allergy remain scarce. One of the most severe chronic forms of fungal disease is allergic bronchopulmonary mycoses, which occur in patients with chronic lung diseases such as COPD or cystic fibrosis. In this collection, Michel et al. demonstrate in 29 adults with cystic fibrosis that basophil and lymphocyte activation tests can enhance the diagnosis of allergic bronchopulmonary mycoses, compared to the usual humoral immunity biomarkers.

Understanding how the immunological machinery of the host and the fungal armory of stress responses interact to dictate the outcome of the host-fungus interaction. Understanding these interactions will provide critical insights into fungal disease mechanisms and pinpointing relevant immune and fungal molecules that ensure protection or drive disease might lead to new therapeutic targets. In this regard, Loureiro et al. explore macrophage extracellular traps (METosis), which are extracellular DNA fibers released by macrophages that are able to entrap and kill various microbes. They observe that upon contact with *C. albicans in vitro*, (i) macrophages phagocytose and engulf yeast cells, (ii) METosis and phagocytosis can occur simultaneously, and (iii) both processes are important in controlling yeast cells proliferation in the first hours of infection, particularly in neutropenic patients (Loureiro et al.). Their findings suggest that yeast extracellular DNase activity might be an important virulence factor, which would degrade the extracellular DNA traps (Loureiro et al.). In another *in vitro* study conducted on *Talaromyces marneffei*, Li et al. unveil a novel pathway by which this opportunistic fungal pathogen may suppress the immune response to its advantage. Their findings show how IL-6, a key factor in acute inflammatory responses, is down-regulated in bronchial epithelial cells infected with *Talaromyces marneffei*. This inhibition occurs via lncSSBP1, a novel long non-coding RNA that specifically interacts with heterogeneous nuclear ribonucleoprotein K, which is involved in IL-6 mRNA processing (Li et al.). Likewise, the two *in vivo* mouse studies published in this collection provide novel insights into host-fungi interactions mechanisms. Amarsaikhan et al. identify a novel role for the adiponectin pathway in the inhibition of lung inflammatory responses to chitin and *A. fumigatus* inhalation. Fa et al. support that TNF-α expression by an engineered *Cryptococcus neoformans* strain is insufficient to drive complete immune protection, but strongly enhance protective responses during primary cryptococcal infection when compared to wild type strain-infected mice.

The incidence of invasive fungal diseases is increasing with a global rise in the number of immunocompromised patients, who have deficient antifungal defense mechanisms that primarily affect the phagocytic activity of immune cells and the overall inflammatory response. Yet, infection by molds and other pathogens induce elevated levels of several cytokines, including IL-6 and IL-8, even in severely immunocompromised patients (Rawlings et al.). In a clinical study, Rawlings et al. find that elevated IL-6 and IL-8 levels in the blood or BAL fluid at the time of bronchoscopy and, perhaps more importantly, rising levels in blood 4 days following bronchoscopy predict mortality in 106 patients with underlying hematological malignancy who underwent bronchoscopy for suspected mold infection.

The high variability in the onset and clinical course of fungal diseases in patients raises fundamental questions about host and fungal factor that are critical in regulating pathogenesis. Exciting approaches aimed at dissecting the respective influence of host and fungus genetic backgrounds on disease severity, including fungal diseases, are rapidly advancing. Puértolas-Balint et al. address the issue of the fungal pathogen-associated factors by comparing the virulence-associated gene (VRG) content in the whole-genome sequence of four *A. fumigatus* isolates from either clinical or environmental origins. They highlight a high genetic diversity among these isolates, with up to 68,352 total number of total genetic variants. In contrast, the genomic VRG content was similar in all isolates, demonstrating that clinical and environmental isolates share the same pathogenic potential, at least at the genome level. However, their comparative genomic analysis highlight the presence of both single nucleotide polymorphisms within VRGs, and repetitive genetic elements located next to VRG groups, which could alter gene regulation and explain heterogeneous virulence phenotypes observed among *A. fumigatus* isolates (Puértolas-Balint et al.). On the flipside of the coin, Danion et al. investigate how the stimulation of neutrophils and peripheral blood mononuclear cells from *STAT3*-deficient patients impacts the immune responses induced by *A. fumigatus*. They find that *STAT3*-deficiency leads to a defective adaptive immune response against *A. fumigatus* infection, characterized by lower IFN-γ and IL-17 responses, and conclude that the potential benefit of IFN-γ treatment in *STAT3*-deficient patients with aspergillosis warrants further study (Danion et al.).

Overall, this themed collection enhances our knowledge of the molecular and cellular processes involved in fungal diseases susceptibility, which paves the way toward personalized medical interventions based on host-directed risk stratification and individualized diagnosis and therapy.

## Author Contributions

SR drafted the manuscript. All authors contributed to manuscript revision, read, and approved the submitted version.

## Conflict of Interest

The authors declare that the research was conducted in the absence of any commercial or financial relationships that could be construed as a potential conflict of interest.

